# Intrinsic hydrophobicity of IDP-based biomolecular condensates drives their partial drying on membrane surfaces

**DOI:** 10.1063/5.0253522

**Published:** 2025-03-17

**Authors:** J. Holland, T. J. Nott, D. G. A. L. Aarts

**Affiliations:** 1 Department of Chemistry, Physical and Theoretical Chemistry Laboratory, University of Oxford, Oxford OX1 3QZ, United Kingdom; 2 Department of Chemistry, King’s College London, Britannia House, 7 Trinity Street, London SE1 1DB, United Kingdom

## Abstract

The localization of biomolecular condensates to intracellular membrane surfaces has emerged as an important feature of sub-cellular organization. In this work, we study the wetting behavior of biomolecular condensates on various substrates. We use confocal microscopy to measure the contact angles of model condensates formed by intrinsically disordered protein Ddx4^N^. We show the importance of taking optical aberrations into account, as these impact apparent contact angle measurements. Ddx4^N^ condensates are seen to partially dry (contact angles above 90°) a model membrane, with little dependence on the magnitude of charge on, or tyrosine content of, Ddx4^N^. Further contact angle measurements on surfaces of varying hydrophilicity reveal a preference of Ddx4^N^ condensates for hydrophobic surfaces, suggesting an intrinsic repulsion between protein condensates and hydrophilic membrane surfaces. This observation is in line with previous studies relating protein adsorption to surface hydrophilicity. Our work advances the understanding of the molecular details governing the localization of biomolecular condensates.

## I. INTRODUCTION

Surfaces inside cells show wetting behavior by biomolecular condensates—functional, liquid-like densities rich in multivalent proteins.^1–8^ This wetting often imparts functionality; for example, sculpting the cytoskeletal network,^3–5,9^ facilitating a production line for ribosome subunits by controlling nucleolar sub-structure,^10^ and mediating interactions at the endoplasmic reticulum.^6^ There are also many cases of biomolecular condensates wetting the plasma membrane,^6–8,11–19^ while other condensates, differing in composition, remain suspended in the intracellular milleu with no obvious surface localization.^2^ Furthermore, recent work suggests that glycinin condensates can remodel membranes, on a geometric and molecular level, through spontaneous wetting.^20,21^ Much work over the past 15 years has been devoted to understanding how the sequences of condensate-associated proteins stabilize their condensates, yet discerning how biology controls condensate wetting is a relatively open question.^22^ The degree of wetting is characterized by the contact angle;^23^ throughout this paper we will use “partial drying” to describe a (internal) contact angle larger than 90°,^24^ “partial wetting” to refer to contact angles below 90°, and increased wetting to refer to decreasing contact angles.

Here, we are concerned with the nature of interactions between condensates and membrane surfaces, a ubiquitous component of living cells. In addition, we are interested in how surface wetting by condensates is modulated by a combination of protein sequence and surface chemistry. These points are motivated by the broader question of the extent to which condensate wetting is driven by the same sequence features which control other condensate properties, such as stability and material state. A clearer understanding would further uncover the design space available for intrinsically disordered protein (IDP)-based condensate functionality, in both physiological and synthetic systems. To this end, we use a reductionist approach, and study the interaction of a canonical model membrane and model biomolecular condensate. The former comprises a 1-palmitoyl-2-oleoyl-*sn*-glycero-3-phosphocholine (POPC) supported lipid bilayer. This phospholipid spontaneously forms bilayers in aqueous solution, exposing its hydrophilic, zwitterionic, headgroup to the surrounding solvent and is commonly used to prepare biomimetic membranes for biophysical studies.^25^ Our model biomolecular condensates are formed by the phase-separation of intrinsically disordered protein (IDP), Ddx4^N^. IDPs are proteins that behave as flexible polymers in solutions exhibiting transient interactions and represent a broad class of condensate associated proteins.^26^ Ddx4^N^ contains sequence features common to many condensate-associated proteins (charge patterning, RGG repeats, and hydrophobic residues)^27–30^ and serves as an established model system for IDP-based biomolecular condensates.^31–36^ Thus, by studying the interaction of Ddx4^N^ condensates with a POPC bilayer, we seek to illuminate general principles governing the behavior of a wide range of biomolecular condensates at membrane surfaces.

We use confocal microscopy to capture images of a series of Ddx4^N^ condensates, altering their charge and tyrosine content (known modulators of IDP phase-separation^28,37,38^), sedimented onto our biomimetic POPC supported lipid bilayer. These images are corrected for aberrations, before the degree of wetting is characterized by fitting the Young–Laplace equation for sessile drops to condensate interfaces, accurately determining their contact angles. All the tested Ddx4^N^ condensates are seen to partially dry on our model membrane. To test whether this partial drying is due to the intrinsically hydrophobic nature of condensates, further Ddx4^N^ condensate contact angle measurements are performed on siliconized glass and untreated glass surfaces. These two surfaces have decreased affinity for aqueous solutions, relative to our model membrane surface, giving insight into the contribution of the hydrophobic force to condensate wetting. By relating our contact angle measurements to phase diagrams for each condensate, we link condensate wetting to their stability and water content. This reveals an intrinsic hydrophobic repulsion between Ddx4^N^ condensates and our model membrane.

This paper is organized as follows: first, we present details of the experimental methods and analysis in this work, including sample preparation and imaging, as well as contact angle measurement through fitting of the Young–Laplace equation (Sec. II). Emphasis is placed on explaining imaging artifacts caused by refraction at condensate interfaces and the point spread function of our optical setup. We present methods to correct for these aberrations, supported by simulations of confocal imaging of sessile condensates. Section III presents the measurements of Ddx4^N^ condensate contact angles on a model membrane, siliconized glass, and untreated glass, along with phase diagrams of each Ddx4^N^ variant (Sec. III). This paper concludes with a contextualization of our results within, and implications for, biomolecular condensate biology (Sec. IV).

## II. EXPERIMENTAL METHODS

### A. Protein expression and purification

Aqueous solutions of Ddx4^N^ used in this work were purified from bacterial cell culture expressing recombinant Ddx4^N^ as follows. BL21(DE3) *E. coli* cells were transformed with IPTG inducible pET-SUMO 2HTb plasmids encoding the desired Ddx4^N^ sequence and grown in autoinduction media^39^ at 37 °C until reaching an optical density of 2. The temperature was then reduced to 18 °C and cells were left to express the Ddx4^N^ construct overnight. Cell pellets were resuspended in buffer (6M guanidinium chloride, 10 mM imidazole, 20 mM sodium phosphate pH 7.4), and lysed by sonication. The His-tagged protein was purified by affinity chromatography using nickel NTA agarose resin (Agarose Bead Technologies). ULP1 was then added to remove the tag, and the eluent containing the target protein was further purified through size exclusion chromatography, simultaneously exchanging the protein product into storage buffer (300 mM NaCl, 20 mM Tris pH 8).

### B. Contact angle measurement through drop shape analysis

#### 1. Sample preparation and imaging

Sessile condensates were prepared for imaging by making an equivolume mixture of recombinantly expressed and purified Ddx4^N^ (80–350 *μ*M) and 5 *μ*M Alexa-488 in 20 mM Tris pH 8 at 50 °C, to give a phase-separating solution above its transition temperature. 9.1 *μ*l of this solution was placed in the center of a coverslip carrying the surface of interest, which was then sealed with a siliconized glass coverslip and 0.12 mm imaging spacer (Sigma). As the sample cooled to room temperature, phase-separation through nucleation and growth formed protein-rich condensates and a protein-poor “buffer phase.” Larger condensates with radii in excess of 10 *μ*m were generated by allowing the phase-separating solution to ambiently cool, nucleating droplets inside the sample tube and inducing coalescence through either leaving samples to sediment naturally for 10 min, or centrifuged for 2 min at 20 000 rcf. Condensates were then aspirated from the base of the sample tube for imaging.

Three-dimensional images of condensates were captured after a 1 h equilibration period using a Leica TCS-SP5 confocal microscope equipped with a HCX PL APO CS 63.0 × 1.40 OIL UV objective. Fluorescent light was imaged using 488 nm incident light, collecting emitted light with wavelengths in the range of 560–630 nm. To accurately determine the position of the base of the sample, we also imaged light reflected by the sample in the range of 483–493 nm. This produced a well-defined intensity at the glass–liquid interface, accurately defining the base of the sample for contact angle measurement. In all the cases, the dichroic mirror used was TD 488/543/633.

To obtain contact angles from our confocal images, we employ axisymmetric drop-shape analysis, where we fit the Young–Laplace equation for sessile drops to the profiles of our sessile condensates. This enables the interpolation of our confocal images between pixels, where the true contact point lies. To do so, we use an automated image analysis protocol developed in Mathematica 13, which operates on our 3D confocal images and returns coordinates describing the axisymmetric profile of our condensates. These are then numerically fitted to the Young–Laplace equation, which defines their contact angle. Throughout Secs. II B 2 and II B 3, the bold font indicates a function or object in Mathematica.

#### 2. Image analysis

**FIG. 1.**
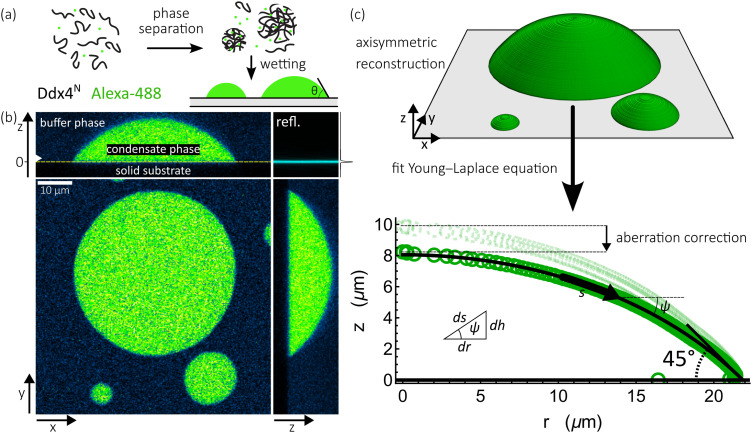
(a) Cartoon showing phase-separation of Ddx4^N^ in the presence of trace amounts of Alexa-488 forms biomolecular condensates enriched in Ddx4^N^ and Alexa-488, which can wet solid substrates. (b) Three-dimensional image of sessile condensates obtained through confocal microscopy; collecting light fluoresced by Alexa-488 and light reflected at the glass surface at the base of the sample. (c) Axisymmetric reconstruction of sessile condensates by measuring mean condensate radius at each height *z* above the surface enables the measurement of best-fit contact angles through fitting of the Young–Laplace equation (see Sec. II B 3). Geometric definitions of variables *s*, *ψ*, *h*, and *r* in Eq. (1) are illustrated. Before fitting to the Young–Laplace equation, profile coordinates are transformed by a vertical factor of 1/1.2 to correct for optical aberrations (see Sec. II B 4).

Masks of condensates are generated from three-dimensional z-stacks, comprising fluorescent and reflected light channels, sections of which are shown in Fig. 1(a). The solid–liquid interface at the base of the sample is defined as the z position with maximum intensity, averaged over *xy* slices. Any *xy* slices below the base are discarded, leaving a 3D image of only sessile condensates. This is subject to a **GaussianFilter** of voxel radius 2, before binarization using Otsu’s algorithm. Masks of condensates are generated using the **SelectComponents** function, storing regions of interconnected voxels not in contact with any surface other than the base as **SparseArray** objects.

Three-dimensional masks of condensates are converted into a set of (*r*, *z*) coordinates describing their axisymmetric profile by first mapping the **Image** function onto a condensate’s **SparseArray**. This returns a **list** of *xy* images of its binary mask. The radius of the drop at each height z is measured by applying the **FillingTransform** function to remove any 0 pixels in the interior of the drop and then using the **ComponentsMeasurements** function to find the radius of the best fit disk. Each radius is then augmented by the (small) difference between the center of the disk and the *xy* coordinates of the droplet centroid, ensuring the radius measured is the mean distance from the optimal symmetry axis of drops. To each set of (*r*, *z*) coordinates, a point at (0, *z*_max_) is appended, where *z*_max_ is the height of the condensate, corresponding to the apex of the drop.

#### 3. Contact angle measurement

Contact angles are extracted from profiles by using Mathematica 13 to optimize the Young–Laplace equation describing their interface, as we have previously employed for capillary length measurement of sessile condensates.^36^ Balancing hydrostatic and Laplace pressures for a sessile drop exhibiting full rotational symmetry about its *z* axis, parallel to a uniform gravitational field, yields the following Young–Laplace equation describing its surface:^40–44^

$$\frac{d\psi }{ds}=\frac{2}{R_{0}}+\frac{h}{\ell_{c}^{2}}-\frac{\sin\psi }{r}.$$ (1)

Here, *s* is the distance along the surface from the apex—defining the origin of coordinates, *ψ* is its angle incident to the horizontal, and *r* is the radius at height *h*. The meanings of variables *s*, *ψ*, *r*, and *h* are illustrated in Fig. 1(c). Furthermore, *R*_0_ is the curvature at the apex of the drop, and *ℓ*_*c*_ is its capillary length. Integration of Eq. (1) over *s* for fixed values of *R*_0_ and *ℓ*_*c*_ generates theoretical drop profiles, which can be compared to measured profiles.

To find the optimum profile, we rewrite Eq. (1) as

$$\frac{d\psi }{ds}=\frac{2}{R_{0}}+\frac{z-z_{0}}{\ell_{c}^{2}}-\frac{\sin\psi }{r},$$ (2)

where we have substituted *z* − *z*_0_ for *h*, allowing for vertical translation of theoretical profiles by altering *z*_0_. This equation is numerically integrated up to *s*_max_ for trial values of *R*_0_, *ℓ*_*c*_, and *z*_0_. The resultant solution is used to generate 1000 trial points in the (*r*, *z*) plane, evenly spaced from 0 to *s*_max_, which are compared to experimentally determined profile coordinates (*R*_*i*_, *Z*_*i*_) through

$$\lambda \left(R_{0},\ell_{c},z_{0},s_{\max}\right)=\frac{1}{N}\overset{N}{\underset{i=1}{\sum }}\left[{\left(R_{i}-r_{i}\right)}^{2}+{\left(Z_{i}-z_{i}\right)}^{2}\right],$$ (3)

where (*r*_*i*_, *z*_*i*_) are the trial points closest to the corresponding experimentally determined point and *N* is the number of experimental coordinates. This objective function is minimized using the **NMinimize** function to implement the Nelder–Mead algorithm in Mathematica 13. During the fitting procedure, *s*_max_ is defined as

$$s_{\max}=1.2\overset{N-1}{\underset{i=4}{\sum }}\sqrt{{\left({\bar{R}}_{i+1}-{\bar{R}}_{i}\right)}^{2}+{\left(Z_{i+1}-Z_{i}\right)}^{2}},$$ (4)

with

$${\bar{R}}_{i}=\overset{7}{\underset{j=1}{\sum }}\frac{R_{j-3}}{7}.$$ (5)

Equation (4) calculates 1.2× the smoothed distance between consecutive coordinates along an experimentally determined profile. Once *R*_0_, *ℓ*_*c*_, and *z*_0_ have been optimized, *s*_max_ is redefined as *z*(*s*_max_) = 0. The best-fit contact angle *θ* is then given by

$$\theta =-\psi \left(s_{\max}\right).$$ (6)

Condensates satisfying the following condition:

$$\frac{\lambda N}{s_{\max}}>0.5\;\text{μm},$$ (7)

are considered to be poorly fit and discarded.

#### 4. Minimizing the impact of aberrations on contact angle measurements

It is well-documented that imaging aqueous media with an oil immersion objective introduces focal shift and axial offset due to aberrant reflection and refraction at the base of the sample.^45–47^ Our situation is further complicated by biomolecular condensates having a differing refractive index to water, evident by their appearance in DIC microscopy,^34^ ability to be optically trapped,^48^ as well as quantitative phase microscopy measurements.^49^ As such, it is not possible to choose an objective that is optimized for imaging the (refracting) fluid surface of condensates, meaning aberrations will always be present. We notice two obvious aberrations in our images. First, with heights well below their expected capillary lengths,^36^ the Ddx4^N^ condensates studied in this work should adopt spherical cap geometries. This is not the case, as seen in Fig. 4(b). Second, below contact radii of roughly 10 *μ*m, condensates show a contact angle that increases with decreasing radius, as shown in Fig. 5(a). To investigate the impact of these aberrations on our contact angle measurements, we compare our real confocal images to simulated images of spherical caps.

Ideally, we would simulate images of sessile condensates by convolving an appropriate spherical cap with the point spread function (PSF) of our optical system. However, the determination of our point spread function is not straightforward. The inhomogeneous refractive indices throughout our optical setup are expected to cause the form of the PSF to have spatial dependence. Measuring the point spread function at every type of environment in our samples poses a significant technical challenge. Instead, to test the effect of aberrant refractions on imaging, we developed a custom ray-tracing algorithm to simulate confocal imaging of spherical caps of differing refractive index, described in detail in Appendix A.

By applying our contact angle measurement procedure to simulated images of spherical caps generated through ray-tracing, we found that apparent contact angles were brought in line with true contact angles by a vertical rescaling of profile coordinates by a factor of 1/1.2. This was the case regardless of whether the condensate refractive index was set to 1.33 or 1.41 [see Fig. 4(b)], representing aqueous solutions dilute and rich in protein, respectively.^50^ This factor has previously been proposed by Hell *et al.* to account for the focal shift caused by imaging aqueous media with refractive index of 1.33 with a 1.4 NA oil immersion objective.^46^

We next hypothesized that the effect of increasing contact angles with decreasing condensate size is due to the resolution limit imposed by the point spread function. To test this, we recorded 3D confocal images of 100 nm fluorescent beads, suspended 5–15 *μ*m above the base of the sample in an agarose hydrogel, using laser settings identical to those used to capture images of our condensates. Imaging beads at this depth allowed us to resolve most of the form of the PSF, while minimizing distortion associated with deep imaging.^46^ The mean of these images, centered on the beads, was taken as a proxy for the PSF, shown as an *xz* projection in Fig. 5(b).

The effect of the PSF on our images was studied by simulating images by convolving our measured PSF with spherical caps of varying size, as detailed in Appendix B. Measuring the contact angles of the generated images using our standard procedure revealed a similar relationship between contact angle *θ* and contact radius *r*, as seen for our Ddx4^N^ condensates (see Fig. 5). The length *r* over which this effect is noticeable is exaggerated in our simulated data [Fig. 5(b)], relative to our real data [Fig. 5(a)]. This is attributed to the increased axial distortion of the PSF as imaging depth increases; the true PSF near the contact point is likely not as diffuse as our measured PSF. However, it would have been inappropriate to convolve our spherical caps with a PSF measured at the base of samples, as half of the imaging volume would have been located within the glass coverslip. Our data suggest as condensates decrease in size toward that of the PSF, they are rapidly distorted, appearing similar to the PSF. To alleviate the impact of this aberration on our primary study, we discarded all contact angle measurements for condensates with contact radii less than 10 *μ*m.

We would like to highlight that the size-dependent impact of the PSF on contact angles bears stark resemblance to that predicted by a line tension *τ* at the three phase contact line.^51^ This enigmatic phenomena causes contact angles to diverge rapidly toward 0° or 180°, depending on the sign (+/−) of *τ*, as the contact radius decreases past the line-tension length *γ*/*τ*.^41,52^ Our imaging apparatus is not sufficient to reject line tension as the cause of this effect, but we believe it to be unlikely.

### C. Supported lipid bilayer preparation

To observe the interaction of biomolecular condensates with cellular membranes in a controlled environment, a model membrane in the form of a supported lipid bilayer (SLB) was prepared. The model membrane is composed of 1-palmitoyl-2-oleoyl-*sn*-glycero-3-phosphocholine (POPC), whose hydrophilic headgroup and hydrophobic tail drives the spontaneous formation of bilayers, manifest in solution as vesicles. We prepare SLBs through vesicle fusion,^53^ where small unilamellar vesicles (SUVs) fuse and rupture atop a solid, hydrophilic support, creating a lipid bilayer.^54^ To do so, we adapted protocols from Refs. 55 and 56, using POPC supplied as 100 mg powder in sealed ampules from Avanti^®^.

#### 1. Preparation of lipid films

20 × 10 ml and a single 20 ml glass phial(s) were washed with isopropanol, dried with nitrogen gas, and desiccated under vacuum for 1 h. To a 100 mg ampule of POPC, 5 ml chloroform was added using a glass syringe previously washed with chloroform, instantly dissolving the lipids to form a clear solution. The POPC solution was then added to the 20 ml phial, with the air in the phial replaced with nitrogen gas and the solution topped up to 20 ml with chloroform and bath sonicated for 5 min. After sonication, 1 ml of POPC solution was added to a clean 10 ml glass phial. The chloroform was evaporated under a moderate flow of nitrogen gas, rotating the phial at a tilt to create a lipid film on the walls of the phial. This was repeated until 20 aliquots containing 5 mg POPC had been prepared. Aliquots were stored under vacuum overnight with a loose lid to remove any moisture and then at −20 °C for longer term storage with the lid screwed tight.

#### 2. Preparation of SUVs

5 mg POPC was dissolved in 5 ml 150 mM NaCl 2 mM CaCl_2_ (SUV buffer) and vortexed until no lipid film was visible on the inner surface of the phial. The lipid solution was freeze-thawed 5× by holding the phial in liquid nitrogen for 1 min and then at 50 °C in a water bath for 7 min to rupture large multilamellar vesicles. The solution was transferred to a clean, plastic 250 ml beaker, before 45 ml of SUV buffer was added. The beaker was placed on ice during probe sonication, performed with a Fisherbrand^®^ Model 705 Sonic Dismembrator with the following process settings: amplitude 55, process time 15 min, pulse-on time 3 s, and pulse-off time 10 s. The sonicate was centrifuged in a 50 ml Greiner Bio-One^TM^ Polypropylene Conical Bottom Test Tube and then the supernatant (POPC SUV solution) was transferred to a fresh 50 ml tube. The air in the tube was replaced with nitrogen gas before storage at 4 °C. SUVs were used within 3 days of preparation.

#### 3. Lipid coating of borosilicate glass

Ten borosilicate glass coverslips (Agar Scientific Ltd. 22 mm No. 1.5) were cleaned with nitrogen gas, isopropanol, nitrogen gas again, and wiped with lens paper (Fisherbrand^TM^). Each surface of the coverslips was treated with an oxygen plasma for 5 min before submersion in 2× Petri dishes containing 25 ml of POPC SUV solution (five coverslips in each dish). The dishes were left on a rocker for 60 min to allow the supported lipid bilayer to form by vesicle fusion. The solution in the Petri dish was exchanged for fresh SUV buffer twice, with 30 min on the rocker between washes, to remove excess SUVs. Coated coverslips were dried with nitrogen gas and placed in a fresh Petri dish before storage under vacuum. SLB-coated coverslips were used within 3 days of preparation. Preparation of SLBs was verified using atomic force microscopy.

## III. RESULTS AND DISCUSSION

### A. Ddx4^N^ condensates partially dry on membrane surfaces in a manner resistant to sequence modulation

**FIG. 2.**
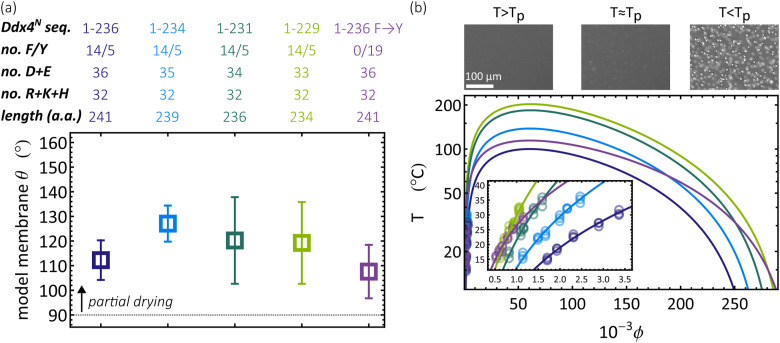
(a) Sequence details and condensate contact angle measurements on the model membrane for Ddx4^N^ variants. (b) Phase diagram measurement through temperature-dependent video microscopy and temperature *T*—volume fraction *ϕ* phase diagrams for Ddx4^N^ variants. Bright-field images for phase diagram measurement were captured using an Olympus BX43 microscope equipped with differential interference contrast optics.

A range of interactions drive the phase-separation of archetypal condensate-forming intrinsically disordered proteins Ddx4, Laf-1, FUS, and hnRNPA1.^28,30,31,57^ These include electrostatic interactions between charged residues, and cation-*π* interactions between arginine and phenylalanine/tyrosine residues. Here, we investigate the effect of sequence specificity on wetting, and its coupling to condensate stability. To do so, we generated a set of mutant Ddx4^N^ proteins, varying the charge and phenyalanine/tyrosine content of a base Ddx4^N^ sequence, where all four native cysteine residues are exchanged for serine, preventing any disulfide bond formation which may induce slow changes in condensate composition. Sequence properties are given in Fig. 2(a), where their names correspond to the section of the base sequence from which it is derived. Shortening the sequence from Ddx4^N^ 1-236 to Ddx4^N^ 1-229 decreases the net negative protein charge at pH 8, while Ddx4^N^ 1–236 F → Y contains a sequence-wide substitution of phenylalanine for tyrosine, known to strengthen cation-*π* interactions.^28,37,38,58^

Data in Fig. 2(a) show that condensates formed by these Ddx4^N^ proteins partially dry our model membrane surface, with all contact angles over 100°. The consistent propensity of partial drying among tested Ddx4^N^ condensates suggests this phenomenon is not controlled by either sequence charge or tyrosine content. To test how these sequence features impacted Ddx4^N^ condensate stability, we constructed phase diagrams [Fig. 2(b)] by recording the onset of phase-separation as a function of temperature—the transition temperature *T*_*p*_, as protein volume fraction *ϕ* is varied.^59^ These data were then fitted to Flory–Huggins theory as previously described,^31,33^ allowing extrapolation of binodals describing the phase behavior of each Ddx4^N^ sequence.

In Fig. 2(b), the widths of condensate binodals are seen to increase in the order Ddx4^N^ 1-236, Ddx4^N^ 1-234, Ddx4^N^ 1-231, and Ddx4^N^ 1-229, revealing that for a more neutral Ddx4^N^, phase-separation is more favored. Furthermore, Ddx4^N^ 1–236 F → Y shows a greater degree of phase-separation than that of Ddx4^N^ 1-236 at the temperature of contact angle measurements (22 °C). This increased condensate stability is attributed to enhanced cation-*π* interactions between Ddx4^N^ chains. Taken together, the data in Fig. 2 suggest that condensates are able to alter their stability, without significantly impacting their partial drying on membranes, with all condensate mutations exhibiting membrane contact angles in excess of 100°.

### B. Ddx4^N^ condensates preferentially wet hydrophobic surfaces

Partial drying of membrane surfaces by Ddx4^N^ condensates indicates that the membrane surface prefers to be in contact with the protein-poor buffer phase. Given that phospholipid membrane surfaces are highly hydrophilic (high affinity for water), and that Ddx4^N^ condensates are depleted in water relative to their coexisting buffer phase, we hypothesize that this partial drying may be due to the expulsion of water from condensates. To test this, we could, in theory, decrease the number of water-repelling hydrophobic residues in Ddx4^N^. However, this is likely to be too detrimental to phase-separation to be experimentally tractable, as hydrophobic residues are canonical drivers of protein phase-separation.^38,60–62^ Instead, we opted to investigate how Ddx4^N^ condensates wet two substrates with decreased water affinity (increased hydrophobicity) relative to our model membrane—(borosilicate) glass and siliconized glass.

**FIG. 3.**
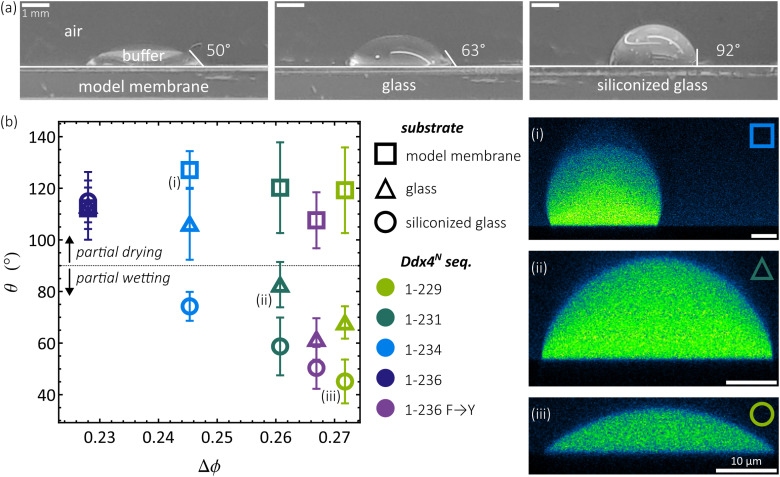
(a) Contact angles of sessile droplets of 150 mM NaCl 20 mM Tris pH 8 buffer, resting on glass-coated with a POPC bilayer (SLB), untreated glass, and siliconized glass. (b) Contact angles of Ddx4^N^ condensates displayed as their mean and standard deviation, plotted against the degree of phase-separation in terms of protein volume fraction Δ*ϕ*. (i)–(iii) *xz* sections of sessile Ddx4^N^ condensates.

**FIG. 4.**
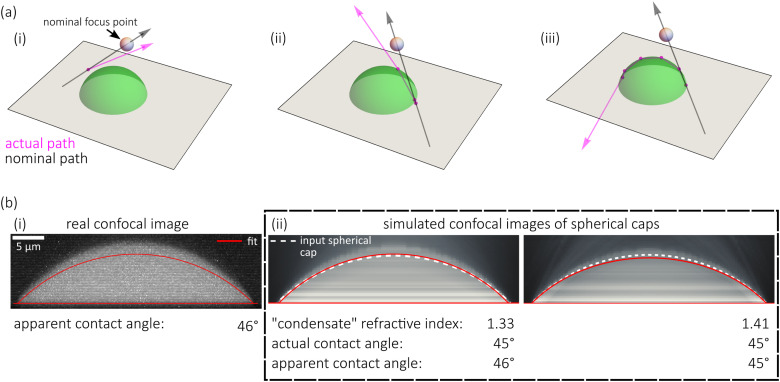
(a) Nominal (black) and actual paths (magenta) of rays with instances of (i) refraction at the substrate, (ii) refraction at the substrate and at the drop surface, and (iii) total internal reflection at the drop surface. The white sphere indicates the nominal focal point. (b) *xz* projections of (i) a real confocal image of a sessile Ddx4^N^ condensate and (ii) images of 45° spherical caps generated through ray-tracing for caps of refractive indices 1.33 and 1.41. The refractive index exterior of the caps, above their base, was set to 1.33 in both cases. The red lines indicate best fit profiles and the dashed white lines indicate the outline of spherical caps used for the simulation of confocal imaging.

**FIG. 5.**
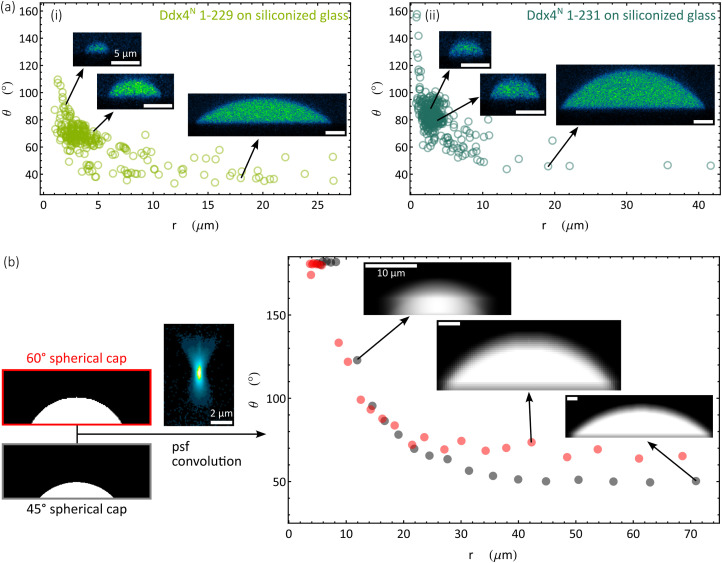
(a) Measured contact angles plotted against their contact radii for (i) Ddx4^N^ 1-229 and (ii) Ddx4^N^ 1-231 condensates resting on siliconized glass. (b) Convolution of spherical caps with the point spread function distorts their apparent contact angle as the size of caps approaches the PSF from above.

To quantify the hydrophobicity of our solid substrates in a manner conducive to subsequent discussion of Ddx4^N^ condensate contact angles on the same surfaces, we measured their in-air contact angles with aqueous buffer (150 mM NaCl 20 mM Tris pH 8.0). This solution comprises >99.7%(v/v) of the protein-poor buffer phase coexisting with condensates, i.e., the same solution without Ddx4^N^. Figure 3(a) shows that their contact angles and hydrophobicity increase in the order 50°, 63°, and 92° for the model membrane, glass, and siliconized glass surfaces, respectively. Using these surfaces, we proceed to investigate how surface hydrophobicity impacts Ddx4^N^ condensate wetting.

In Fig. 3(b), we show the contact angles *θ* of our Ddx4^N^ condensates measured on our model membrane, glass, and siliconized glass, plotted against the degree of phase-separation Δ*ϕ*. We define Δ*ϕ* as the difference between the volume fraction of protein inside condensates and the volume fraction of protein in the surrounding buffer phase, taken from the binodals in Fig. 2(b). This permits the assessment of contact angle in terms of the condensate’s stability, in terms of Δ*ϕ*, and water content, which is given within 2 s.f. by 1 − Δ*ϕ*. With the highest water content, Ddx4^N^ 1-236 showed partial drying on all the tested surfaces, while Ddx4^N^ 1-234 partially dried glass to a lesser extent than the model membrane and partially wet (*θ* < 90°) siliconized glass. Ddx4^N^ 1-231, Ddx4^N^ 1-229, and Ddx4^N^ 1–236 F → Y partially dried the model membrane and partially wet glass and siliconized glass. The degree of partial wetting is seen to increase with surface hydrophobicity (from glass to siliconized glass) and as condensates expel water with increasing Δ*ϕ*, as protein–protein interactions become more favorable. We note that Ddx4^N^ 1–236 F → Y falls on the trend produced by the Ddx4^N^ charge mutants remarkably well, supporting the notion that increased affinity for hydrophobic surfaces is a result of decreasing water content inside condensates, rather than due to a specific sequence feature.

Taken together, our data suggest there is a rough threshold value of Δ*ϕ* above which a condensate switches from partial drying to partial wetting. This threshold value of Δ*ϕ* in turn seems to depend on the affinity of the surface for aqueous buffer, as quantified by its in-air contact angle, with a greater value of Δ*ϕ* required for surfaces with lower in-air buffer contact angles. It may be that the Ddx4^N^ condensates partially dry membrane surfaces, regardless of their degree of phase-separation (Δ*ϕ*), as expulsion of water from condensates always accompanies phase-separation. In this sense, proteinaceous condensates may be considered to be intrinsically hydrophobic. With this in mind, we considered whether the intrinsically hydrophobic nature of proteinaceous condensates might be a general principle inhibiting their wetting of membranes. To make progress on this assertion, we evaluate our results in context of the wider literature on protein–surface interactions.

### C. Intrinsic hydrophobicity of condensates inhibits their wetting of highly hydrophilic membrane surfaces

Previously, Schwierz *et al.*^63^ have investigated correlations between peptide–surface interactions and the hydrophilicity of the surface. They found that the adsorption of both hydrophilic and hydrophobic peptides to solid surfaces is greatly inhibited when their water-in-air contact angle decreases below 50°–60°. This is shown to be due to preferential solvation of these hydrophilic surfaces by water, whereas cases of peptide adsorption is driven by the release of water—the entropic hydrophobic effect. Their reported crossover threshold of the water-in-air contact angle, below which adsorption is disfavored, is in close agreement with the established Berg limit of ∼60°.^64,65^ When water-in-air contact angles are below the Berg limit, water entropy at the solid surface becomes greater than in bulk water, driven by an increased density of possible polar and hydrogen bonding interactions at the surface affording more water configurations than in the bulk.^66^ Thus, as a surface’s water contact angle decreases past 60°, the release of water from the solid–liquid interface into the bulk accompanying protein adsorption, becomes entropically unfavorable. This manifests as a repulsive force, pushing hydrophobic species away from the interface.

To relate this to surface wetting by proteinaceous condensates, we recall that adsorption is driven by the same thermodynamic forces as wetting.^67,68^ The non-specific partial drying of our biomimetic POPC bilayer by Ddx4^N^ condensates is thus directly related to its in-air contact angle with aqueous buffer (50°) being below the Berg limit. Constituent protein molecules within Ddx4^N^ condensates are, therefore, unable to outcompete water for the model membrane’s surface. Conversely, on the glass and siliconized glass surfaces, with in-air aqueous buffer contact angles of 63° and 92°, respectively, hydrophobic release of water by protein adsorption is favored and wetting increases with the condensate protein content.

The requirement for displacement of water accompanying membrane wetting by protein condensates is supported by the recent work of Mangiarotti *et al.*, where they show a dehydration of DOPC membranes by glycinin condensates.^20^ This is consistent with the observation that specific membrane-binding domains are required to traffic proteins to membrane surfaces in cells.^69^ Furthermore, our proposed intrinsic repulsion between (intrinsically hydrophobic) protein condensates and necessarily hydrophilic membrane surfaces is supported by multiple in-cell observations, where condensate adhesion to membranes is augmented by interactions with membrane-bound proteins.^6–8,11–19^ Being below the Berg limit is likely an intrinsic property of biological membranes. The associated intrinsic repulsive force between membrane surfaces and biomolecular condensates likely affords biology more control over membrane localization, involved in numerous cellular processes such as autophagy,^18^ formation of the cortical cytoskeleton,^8^ and stabilizing synaptic function in the nervous system.^11^

## IV. CONCLUSION

We have observed that the contact angle of Ddx4^N^ model biomolecular condensates is lower on more hydrophobic surfaces, where a surface’s hydrophobicity is quantified by its water-in-air contact angle. In addition, the wetting of condensates on hydrophobic surfaces is increased by amino acid mutations, which stabilize condensates, increasing the degree of phase-separation, and volume fraction of protein inside condensates. Therefore, as the water content inside condensates decreases, they preferentially wet more hydrophobic surfaces. Contact angles of all Ddx4^N^ condensates tested on a model membrane, comprising a POPC supported lipid bilayer, were above 100°. This consistent propensity for partial drying is explained by examining the water-in-air contact angle of our model membrane, which was found to be below the Berg limit.^64,65^ In this limit, protein adsorption at the surface has been previously shown to be thermodynamically unfavorable,^66^ regardless of protein sequence.^63^ Our observations are, therefore, in line with previous studies examining forces driving single-molecule adsorption of proteins and extend their conclusions to the wetting behavior of protein condensates. These findings advance the physical understanding of condensate wetting, highlighting the governing role of water in mediating an intrinsic repulsion between condensates and membranes, and attraction between condensates and hydrophobic surfaces.

This study focused on a simple model membrane comprising POPC, a neutrally charged phospholipid. Further work on condensates at membranes with a more complex structure, incorporating diverse lipids, membrane proteins, and sterols, may indeed reveal partial wetting. In this case, the hydrophobic repulsion between condensates and membrane surfaces is anticipated to be overcome by, for example, favorable electrostatic condensate–membrane interactions. Biomolecular condensate hydrophobicity has been previously invoked to describe the multiphase architecture of the nucleolus, with the more hydrophobic condensate phase residing on the interior of the nucleolus.^10^ In light of our findings, we speculate that such multiphase architectures may be generally controlled by tuning condensate hydrophobicity, through altering the strength of condensate-forming interactions and the degree of phase-separation, without significantly impacting condensate–membrane interactions.

The experimental methodology used in this work demonstrates the need for careful consideration of optical aberrations associated with confocal microscopy of biomolecular condensates. We observed distortions of droplet geometry, originating from (i) refractive index mismatch between the immersion media and sample, present in all condensates, and (ii) the point spread function of our setup, manifest as the size of condensates approached that of the point spread function. The former was accounted for in contact angle measurements by axial rescaling of our images, while the effect of the latter was absolved by discarding images of smaller condensates.

## Data Availability

The data that support the findings of this study are available from the corresponding authors upon reasonable request.

## APPENDIX A: RAY TRACING

In the ideal case of confocal imaging, all light paths from the objective to its (nominal) focal point would be unaltered by the sample. Our ray tracing algorithm calculates the reflection and refraction of otherwise focused light by the condensate and surrounding solution. These rays are then used to calculate the intensity of fluorescent light collected at a given nominal focal point. Images are generated calculating the intensity for a collection of nominal focal points, which spans the volume of our drop.

The intensity of a point **r** in a fluorescent image is given by

$$I\left(r\right)=\int_{\text{all space}}I_{\text{f}}\left(r,r^{\prime }\right)\Phi \left(r,r^{\prime }\right)\;dr^{\prime },$$ (A1)

where *I*_f_(**r,r′**) is the fluorescence intensity density at point **r′** and Φ(**r,r′**) describes the probability that a photon emitted at point **r′** is focused onto the photomultiplier tube by the objective lens. The fluorescence intensity density is given by

$$I_{f}\left(r^{\prime },r\right)=I_{\text{excitation}}\left(r^{\prime },r\right)\alpha \left(r^{\prime }\right),$$ (A2)

where *I*_excitation_(**r′,r**) is the excitation density and *α*(**r′**) gives the probability of a photon absorption and subsequent fluorescence at point **r′**. We relate the excitation intensity to the intensity of the illumination source through

$$I_{\text{excitation}}\left(r^{\prime }\right)=I_{\text{laser}}\Phi^{\prime }\left(r,r^{\prime }\right),$$ (A3)

where Φ′(**r**) gives the probability that an incident photon will arrive at point **r**. In a confocal microscope, the pinholes are conjugate to the nominal plane, thus Φ′(**r**) = Φ(**r**). Equation (A1) then becomes

$$I\left(r\right)=\int_{\text{all space}}\left(I_{\text{laser}}\Phi \left(r,r^{\prime }\right)\alpha \left(r^{\prime }\right)\right)\Phi \left(r,r^{\prime }\right)\;dr^{\prime }.$$ (A4)

When imaging an ideal sample, the index matched to the objective lens everywhere, Φ(**r,r′**) is given by the point spread function (PSF) of the imaging setup, and is uniform everywhere. Our goal is to calculate how Φ(**r,r′**) is affected by the refractive index mismatch between the objective lens, sessile condensates, and their surrounding fluid phase. This is done discretely through a ray tracing scheme outlined in the following.

We take our sample to consist of three regions of differing refractive index, divided by a spherical cap intersecting a plane at its base, representing a sessile condensate resting on glass. The refractive index of the glass is taken to be 1.51, which outside the condensate is 1.33 and inside the condensate 1.41. The paths of rays incident from the objective lens nominally focused at a given point are procedurally calculated and reported as

$$L=\left(\ell_{i,1},\ell_{i,2}\ldots \ell_{i,j}\right),$$ (A5)

with each line segment defined as

$$\ell_{i,j}=u_{i,j}+\lambda v_{i,j},$$ (A6)

where **u**_**i**_ is the intersection point of the (*i* − 1)th segment with a refracting interface and **v**_**i**_ is a unit vector pointing in the direction of the refraction/reflection calculated according to Snell’s law. The set of vectors {**u**_*i*,1_} are drawn from points on a spherical cone with solid angle 1.4—the numerical aperture of our objective lens, whose apex defines the nominal focal point **r**. {**v**_*i*,1_} are the corresponding unit vectors pointing toward the nominal focal point. The calculation of ray segments terminates when the terminal segment points away from all the refracting interfaces. Figure 4(a) shows the nominal and actual paths of the three rays, illustrating the effects of refraction and reflection on the light path. The intensity of each ray segment is also calculated using Fresnel’s equations, with initial segments taken to have unit intensity. Finally, we discard segments and intensities corresponding to paths outside the sessile condensate, equivalent to setting *α* to 1 inside the condensate and 0 otherwise.

To calculate the integral in Eq. (A4), we divide our sample into a hexahedral mesh. An intersection matrix **X** is calculated with elements,

$$x_{i,j}=\left\{\begin{array}{cc} 1\; & \text{if ray segment }i\text{ intersects volume element }j,\\ 0\; & \text{otherwise.} \end{array}\right.$$ (A7)

The sum of each row gives the number of rays intersecting each corresponding volume element, yielding vector **Φ** whose basis is the sample-spanning mesh. The factor *I*_laser_Φ(***r,r′***) is given by multiplying **X** by the column vector containing the intensities of each ray segment, and taking the total of each row. This vector gives the fluorescence intensity ***I***_f_ in the basis of the sample-spanning mesh. Equation (A4) is then approximated by

$$I\left(r\right)=I_{\text{f}}\left(r\right)\cdot \Phi \left(r\right).$$ (A8)

Images of sessile drops are calculated by scanning a plane of lattice points, which cover half of a cross section of a sessile drop. Two *xz* images of spherical caps with contact angles of 45°, and refractive indices of 1.33 and 1.41, generated through ray tracing, are shown in Fig. 4(b-ii). This algorithm focuses on the geometrical features of electromagnetic radiation, ignoring its wave-like nature. A more involved calculation would incorporate interference between intersecting rays; however, this will not alter the essential features that we are trying to capture. We note that we also ignore reflections accompanying refraction; however, their diminished intensity is not expected to not substantially alter our generated images. Our ray tracing was also found to reproduce axial distortion, presented by Besseling *et al.*^47^ found when imaging homogeneous media of refractive index 1.41 and 1.33 using a 1.4 NA oil objective.

## APPENDIX B: CONVOLUTION OF SPHERICAL CAPS WITH OUR MEASURED PSF

To test aberrations introduced by the form of the point spread function (PSF) in our confocal imaging system, we convolved spherical caps with our measured PSF as follows. Spherical caps of 45° and 60° were generated as Image3D objects in Mathematica 14. These were convolved with rescalings of the PSF, generating a series of convolved caps, which were then treated as real condensate images for the measurement of their contact angle. During fitting, the dimensions of the convolved caps were converted to real space by appropriate scaling with respect to the resolution of the point spread function. Figure 5(b) illustrates this process, showing *xz* projections of 45° and 60° spherical caps and the PSF. The plot in Fig. 5(b) shows that convolving the PSF with spherical caps increases their apparent contact angle *θ* as their apparent contact radius *r* decreases, with *xz* projections inset.
